# Multidisciplinary Challenges in Mastocytosis and How to Address with Personalized Medicine Approaches

**DOI:** 10.3390/ijms20122976

**Published:** 2019-06-18

**Authors:** Peter Valent, Cem Akin, Karoline V. Gleixner, Wolfgang R. Sperr, Andreas Reiter, Michel Arock, Massimo Triggiani

**Affiliations:** 1 Department of Internal Medicine I, Division of Hematology & Hemostaseology, Medical University of Vienna, 1090 Vienna, Austria; karoline.gleixner@meduniwien.ac.at (K.V.G.); wolfgang.r.sperr@meduniwien.ac.at (W.R.S.); 2 Ludwig Boltzmann Institute for Hematology and Oncology, Medical University of Vienna, 1090 Vienna, Austria; 3 Division of Allergy and Clinical Immunology, University of Michigan, Ann Arbor, MI 48106, USA; cemakin@umich.edu; 4 III. Medizinische Klinik, Universitätsmedizin Mannheim, 68167 Mannheim, Germany; andreas.reiter@uum.de; 5 Department of Hematological Biology, Pitié-Salpêtrière Hospital, Pierre et Marie Curie University (UPMC), 75005 Paris, France; arock@ens-cachan.fr; 6 Division of Allergy and Clinical Immunology, University of Salerno, 84131 Salerno, Italy; mtriggiani@unisa.it

**Keywords:** mast cells, *KIT* D816V, tryptase, IgE, allergy, MCAS, personalized medicine

## Abstract

Mastocytosis is a hematopoietic neoplasm defined by abnormal expansion and focal accumulation of clonal tissue mast cells in various organ-systems. The disease exhibits a complex pathology and an equally complex clinical behavior. The classification of the World Health Organization (WHO) divides mastocytosis into cutaneous forms, systemic variants, and localized mast cell tumors. In >80% of patients with systemic mastocytosis (SM), a somatic point mutation in *KIT* at codon 816 is found. Whereas patients with indolent forms of the disease have a normal or near-normal life expectancy, patients with advanced mast cell neoplasms, including aggressive SM and mast cell leukemia, have a poor prognosis with short survival times. In a majority of these patients, multiple somatic mutations and/or an associated hematologic neoplasm, such as a myeloid leukemia, may be detected. Independent of the category of mastocytosis and the serum tryptase level, patients may suffer from mediator-related symptoms and/or osteopathy. Depending on the presence of co-morbidities, the symptomatology in such patients may be mild, severe or even life-threatening. Most relevant co-morbidities in such patients are IgE-dependent allergies, psychiatric, psychological or mental problems, and vitamin D deficiency. The diagnosis and management of mastocytosis is an emerging challenge in clinical practice and requires vast knowledge, a multidisciplinary approach, and personalized medicine procedures. In this article, the current knowledge about mastocytosis is reviewed with special emphasis on the multidisciplinary aspects of the disease and related challenges in daily practice.

## 1. Diagnosis and Classification of Mastocytosis

Mastocytosis is a group of myeloid neoplasms characterized by expansion and focal accumulation of clonal mast cells (MC) in various organ systems, including the skin, the bone marrow (BM) and other internal organs [1,2,3,4,5,6]. Skin lesions are found in most patients. In general, mastocytosis can be divided into cutaneous mastocytosis (CM) where only the skin is affected and systemic mastocytosis (SM) where one or several internal organs, including the BM, are involved [1,2,3,4,5,6,7,8,9,10]. Whereas most patients with CM are children, most adult patients are suffering from SM. CM can further be divided into: (i) maculopapular CM (MPCM), also known as urticaria pigmentosa (UP); (ii) diffuse cutaneous mastocytosis (DCM); and iii) localized mastocytoma of skin [1,2,3,9,10]. In patients with CM, criteria for SM are not fulfilled even if clonal MC can be detected in extracutaneous organs [9,10]. The major criterion of SM is the multifocal accumulation and clustering of MC (at least 15 MC/cluster) in the BM or in another extra-cutaneous organ [9]. Minor SM criteria include an abnormal morphology of MC (spindling, hypogranulation, cytoplasmic extensions); expression of CD2 and/or CD25 in MC in the BM or in (an)other extracutaneous organ(s); expression of an activating mutation in codon 816 of *KIT* (usually *KIT* D816V) in the BM or in (an)other extra-cutaneous organ(s); and a basal serum tryptase level exceeding 20 ng/mL [9]. When the major SM criterion plus at least one minor SM criterion or at least 3 minor SM criteria are fulfilled, SM can be diagnosed (Table S1) [9].

During the past 20 years, an international (EU/US) consensus consortium, including members of the European Competence Network on Mastocytosis (ECNM) and members of the recently established American Initiative for Mast Cell Disorders (AIM), have developed and have repeatedly refined diagnostic criteria, standards, tools and approaches for the diagnosis, classification and management of mastocytosis [9,10,11,12,13,14,15,16]. The diagnostic criteria and the related classification, proposed by this EU/US (ECNM/AIM) consensus group, were adopted by the World Health Organization (WHO) [16,17,18]. In addition, members of this consensus group have established guidelines for the diagnosis, management, therapy and prognostication of patients with mastocytosis [9,10,11,12,13,14,15,16,17,18,19,20]. The resulting diagnostic and management-algorithms are widely accepted.

Recommended clinical and laboratory parameters to investigate in patients with suspected SM include a thorough physical examination, a complete blood count with differential counts, serum chemistry including a basal serum tryptase level, and molecular studies to detect or exclude *KIT* D816V in blood leukocytes [20]. In adults with suspected SM, a thorough BM investigation is recommended when one or more of the following parameters are identified: typical skin lesions (mastocytosis in the skin); a clearly elevated basal serum tryptase level; a *KIT* mutation (usually *KIT* D816V); and/or typical clinical sign or symptoms that have no known etiology, such as unexplained osteoporosis (especially in men), unexplained lymphadenopathy and/or splenomegaly, or unexplained repeated anaphylactic episodes after bee or wasp stings (Table 1) [1,2,3,4,11,13,14,15,16,17,18,19,20]. An accumulation of such findings increases the likelihood of SM. A diagnostic algorithm, incorporating these parameters, has been published recently [20].

According to the WHO classification, SM can be divided into: indolent SM (ISM); smoldering SM (SSM); SM with an associated hematologic (non-MC-lineage) neoplasm (SM-AHN); aggressive SM (ASM); and MC leukemia (MCL) (Table S2) [3,4,9,14,15,16,17,18]. SM-AHN, ASM and MCL are collectively referred to as advanced SM (Table 2). BM mastocytosis (BMM) is a provisional sub-variant of SM where MC infiltration is largely restricted to the BM, the tryptase level is low or normal, and no skin lesions can be detected [9,14,15,16,17,18]. MC sarcoma (MCS) is a highly aggressive variant defined by a local accumulation and invasive (sarcoma-like) growth of highly atypical, neoplastic MC without systemic involvement [1,9,14,15,16,17,18,21]. The prognosis in these patients is poor. Most cases progress to MCL within a short time [9,21]. An intriguing aspect is that in advanced SM, skin lesions are often absent, which may lead to a delay in diagnosis and therapy, and may thus result in a less favorable outcome [1,9,14,15,16,17,18].

A number of different studies have validated and confirmed the prognostic impact of the WHO classification regarding survival in CM and SM, as well as in SM variants [13,22,23,24,25,26]. Patients with CM, BMM and ISM have a normal or near normal life-expectancy. In patients with SSM, the prognosis is less favorable because some of these patients progress to advanced SM. In patients with advanced mastocytosis (SM-AHN, ASM, MCL, MCS), the prognosis is poor and the survival is usually short [1,9,14,15,16,17,18,22,23,24,25].

In all patients with mastocytosis, regardless of variant, age, and other variables, mediator-induced symptoms and/or an osteopathy can develop [2,3,9,14,15,16,17,18,27,28,29,30]. Depending on the presence of co-morbidities, the symptomatology in such patients may be mild, severe or even life-threatening. Relevant co-morbidities in such patients are IgE-dependent allergies, psychiatric or psychological problems, and vitamin D deficiency (Table 3). However, other co-morbidities, such as infections, chronic inflammation or food intolerance, may also aggravate mediator-induced symptoms in patients with mastocytosis.

During the past few years, knowledge about the pathogenesis of mastocytosis has increased substantially [30,31,32,33,34,35,36,37,38,39]. In addition, new treatments have been developed to block mediator secretion, mediator effects and/or the growth of neoplastic MC [40,41,42,43,44,45,46,47,48]. However, not all patients may benefit from these therapies and there is still a need to improve prognostication and management. In particular, patients with multi-organ involvement and co-morbidities, it is often difficult to establish an optimal management plan. Moreover, although more and more cases are diagnosed, mastocytosis is still regarded a rare disease with a complex pathology and an unpredictable clinical course. The diagnosis and management of MC neoplasms is an emerging challenge and requires vast knowledge, a multidisciplinary approach and personalized medicine-based procedures. In the current article, the current knowledge about mastocytosis is reviewed with special emphasis on the multidisciplinary aspects of the disease and related challenges in daily practice. 

## 2. Hematologic Prognostication

Patients with ISM have an excellent prognosis because the rate of progression to high-grade (advanced) SM is low [22,23,24,25,26]. However, some of these patients may finally progress to SM-AHN, ASM or MCL. A number of prognostic variables predicting progression of ISM/SSM to a high-grade disease have been identified. These include, among others, multi-lineage involvement of hematopoietic cells with *KIT* D816V, the variant allele frequency (VAF) of mutated *KIT*, an elevated β2-microglobulin, elevated alkaline phosphatase levels, lymphadenopathy and splenomegaly, and the presence of additional somatic mutations (Table 4) [22,23,24,25,37,38,39,49,50,51]. Several of these parameters (B-findings) are indicative of SSM [3,9,16,17,18,52]. In patients with advanced SM, the type of SM is also of prognostic significance. In particular, patients with MCL have a poor prognosis with reduced survival compared to those with ASM [22,23,24,25]. Moreover, patients with ASM can be split into those with slow progression and those with rapid progression. In ASM patients with rapid progression, serum tryptase levels increase rapidly and multi-organ damage occurs (or worsens) within short time. In these cases, the percentage of MC in BM smears is of major prognostic significance and is usually elevated substantially. Notably, ASM patients with a MC count exceeding 5% have a worse outcome with reduced survival and progression-free survival compared to patients with <5% MC in BM smears (Table 4) [13,53]. As rapid progression to MCL seen in most of these cases, ASM with ≥5% MC in BM smears is collectively termed ASM in transformation (ASM-T) [53]. Other indicators of a poor prognosis are a relatively fast and steadily increasing basal serum tryptase level, a relatively rapid increase in alkaline phosphatase and/or progressive cytopenia (Table 4). Furthermore, the presence of mutations in additional critical target genes (apart from *KIT*), such as *SRSF2*, *ASXL1*, *RUNX1*, or *RAS* is associated with progression and with a poor outcome [39,51]. In particular, the S/A/R panel (*SRSF2*, *ASXL1*, *RUNX1*) is associated with poor prognosis [39,51]. These mutations cluster in patients with SM-AHN and are often detectable in the AHN portion of the neoplasm, and sometimes in both the SM and the AHN component of the disease. Based on this notion, molecular profiling by next generation sequencing (NGS) is a new emerging molecular tool in patients with SM. Since these mutations cluster in patients with advanced SM and are indicative of a poor prognosis, NGS results will soon be integrated in diagnostic and prognostic algorithms and scoring systems in SM. 

There are also prognostic parameters that can help predict responses to certain therapies. For example, ASM patients with signs of rapid progression are usually not responding to interferon-alpha (IFN-A) therapy and only a few of these patients respond to monotherapy with cladribine or midostaurin. Whereas slowly progressing patients without codon 816 mutations in *KIT* may respond well to imatinib or masitinib, this is usually not the case in SM patients in whom neoplastic cells display *KIT* D816V which confers resistance [54,55]. The best response to midostaurin is seen in patients in whom the clone is slowly progressing and is largely dependent on KIT D816V activity (SSM, chronic MCL), whereas in patients with advanced multi-mutated SM, treatment responses are usually poor and/or short-lived (transient) [44,56,57,58]. In these patients, the midostaurin-resistant or relapsing (sub)clone may be *KIT* D816V-negative which can be explained best by a negative selection process [58]. Overall, treatment responses are poor in patients with rapidly progressing ASM (ASM-T) and MCL, independent of the type of therapy and age. Many of these patients exhibit the S/A/R gene panel or mutations in other critical driver genes. In those with ASM, ASM-T and full-blown MCL, poly-chemotherapy and subsequent allogeneic hematopoietic stem cell transplantation (HSCT) may lead to complete remission [59]. However, not all patients can be cured by HSCT. In addition, most patients are not eligible for HSCT because of advanced age or comorbidities. The remission rate is substantially higher in patients with ASM and SM-AHN compared to patients who are suffering from (acute) MCL [59]. In addition, the outcome after HSCT is better and more durable in those who did respond to previous cytoreductive therapy or have stable diseases. In each case, the risk of relapse and progression has to be balanced against side effects and the risk of transplant-related mortality when discussing HSCT in patients with advanced SM. An open question is whether patients with additional mutations (apart from *KIT* D816V) have a worse outcome after HSCT compared to patients without such mutations.

## 3. The Risk of Anaphylaxis and Mast Cell Activation Syndromes (MCAS)

Patients with SM are at high risk of developing severe anaphylactic reactions to various exogenous substances (triggers/allergens) independent of the category of SM, serum tryptase level or organ involvement [5,6,8,14,27,28,29,30]. Sometimes, the reaction is predictable to a degree, whereas in other cases, the trigger(s) cannot be identified and thus the reaction can occur at any time [27,28,29,30,60,61,62]. The most relevant co-morbidity in SM regarding anaphylaxis is an IgE-dependent allergy (Table 3) [60,61,62]. In particular, allergies against bee or wasp venom can induce recurrent severe anaphylactic (often life-threatening) reactions [60,61,62]. In these patients, it is of utmost importance to establish the correct diagnosis, to follow the course of the patients, and to establish a robust management plan. 

The first key questions to answer in cases with suspected severe MC activation and thus MCAS are whether the patient is suffering from a severe IgE-dependent allergy, whether the patient is suffering from a true MCAS and whether mediator-targeting drugs alone are sufficient to stabilize the condition [63,64,65]. In fact, MCAS are rare, but are severe and often manifest as life-threatening events. These patients develop hypersensitivity reactions that are: (i) severe; (ii) systemic (involving at least 2 organ systems); and (iii) recurrent [65,66,67,68]. True MCAS is defined by MCAS criteria which include a diagnostic acute, event-related increase in the serum tryptase level above baseline following the 20% + 2 formula (within a 2–4 h time interval after the event); typical clinical symptoms of anaphylaxis, usually with severe hypotension; and a response of clinical symptoms to drugs targeting mediator effects, mediator production or MC activation [66,67,68,69].

MCAS can be classified into: a) primary MCAS, also known as monoclonal MCAS (=MMAS) where clonal *KIT*-mutated MC are found; b) secondary MCAS where an IgE-dependent allergy (most cases) or another reactive inflammatory disease process is present (and is considered to be the causative etiology); and c) idiopathic MCAS where neither clonal MC nor an IgE-dependent allergy or another underlying condition/disease can be documented [66,67,68,69]. Patients with SM often suffer from a combination of a primary MCAS and secondary MCAS (combined/mixed MCAS) which is a high-risk situation regarding the development of severe anaphylactic reactions. These patients usually require profound anti-allergic treatment, including immunotherapy, glucocorticosteroids, other mediator-targeting drugs, and/or anti-IgE-based (IgE-depleting) drugs (omalizumab) [65,66,67,68,69]. Therefore, it is of major clinical importance to define the underlying disease, such as SM, and to clarify whether the patient is suffering from a primary MCAS (only) or from a primary MCAS accompanied by an IgE-dependent allergy, and thus also from secondary MCAS. An unresolved question is why many patients with SM never develop any mediator-related symptoms despite their massive burden of neoplastic MC. Another unresolved question is whether other mediators, apart from tryptase, can also be employed as MCAS-specific criteria when increasing above the baseline. It also remains undefined whether somatic mutations in myeloid driver genes can augment MC activation. Finally, it remains unknown whether SM patients with extra copies of the alpha tryptase gene (hereditary alpha-tryptasemia) have a higher chance to develop MCAS compared to those without tryptase gene replications. 

## 4. Osteopathy in Mastocytosis: Diagnosis, Prognostication and Challenges

Osteopenia and osteoporosis are frequently encountered in patients with SM [70,71,72,73]. Therefore, an osteodensitometry (T-Score) is a standard diagnostic test in all patients with SM (at diagnosis and during follow up) regardless of age, sex, or symptoms [14,67]. Repeated testing of the T-Score is recommended even in asymptomatic cases. The primary therapeutic goal in SM is to avoid osteoporosis whenever possible [14]. Thus, prophylactic bisphosphonates are recommended as soon as the T-Score drops to below −2 [14]. In female patients and in patients receiving long-term corticosteroids, the risk to develop osteoporosis in SM is very high (Table 3). The risk may further increase with an untreated vitamin D deficiency or/and alcohol consumption. Indeed, a vitamin D deficiency is regarded as an important co-morbidity in the context of SM-mediated osteopathy (Table 3). Obesity is not a risk factor for the development of osteoporosis per se unless the patient is immobilized by the obesity, but is primarily a risk factor for osteoporosis-mediated bone fractures, such as vertebral fractures (Table 3).

In advanced SM, osteolyses may be detected by x-ray studies [3,9,14]. Rarely, these osteolyses are large and contain a clinically relevant MC infiltrate [74]. In these patients, local bone pain may be the leading symptom or even the initially diagnostic sign (when no skin lesions are present). These patients are at high risk of developing bone fractures and of developing disease-progression. In order to confirm the diagnosis in these patients, a local biopsy is usually required. The involved tissue may show a local MC infiltrate (often composed of immature MC) confirming the diagnosis and variant, i.e., advanced SM. These patients are usually treated with intensive cytoreductive agents or IFN-A. In addition, treatment with bisphosphonates (as adjunct to disease-modifying treatment) is usually recommended in patients who develop large-sized osteolyses. More frequently, osteolyses are small-sized and not relevant clinically. Such small-sized lesions can be detected in all variants of SM, including ISM, and do not count as a C-Finding (indicative of advanced SM). It is important to state, that computed tomography—magnetic resonance imaging, scintigraphy, or positron emission tomography (PET) studies—may sometimes add to the staging in SM, but are not standard in the evaluation of osteopathy. More recently, several bone-related cytokines, such as interleukin-6 (IL-6) or RANKL, were found to be elevated and thus indicative of bone loss in SM [75]. However, it remains unknown whether these cytokines trigger the development of bone loss (osteopenia/osteoporosis) in these patients. 

## 5. Management of Advanced SM and of Hematologic Progression

From a hematologic point of view, the most relevant questions are whether the patient has or develops advanced SM and what treatment can be offered. Although the risk is relatively low, patients with ISM may progress to ASM, SM-AHN or MCL [22,23,24,25,39,40,76]. In high risk patients with ISM, a closer monitoring is recommended (3-6 months intervals), and in the case of suspected progression (e.g., platelet drop, development of ascites, progressive organomegaly or/and increase in alkaline phosphatase), a re-investigation of the BM, of all molecular markers, and of all other disease-related parameters should be performed [14,20].

The next critical question to answer is whether the patient is eligible for and would agree to allogeneic HSCT [59,77,78]. In patients with slow progression, HSCT may be delayed and treatment with cladribine (2CdA) or a KIT tyrosine kinase blocker, such as midostaurin, may be initiated (Table 5) [78,79,80,81,82]. In fact, both types of drugs can induce major clinical responses with improvement or disappearance of one or more C-findings, a decrease in the BM MC burden and of serum tryptase levels, a decrease in organomegaly and a decrease in mediator-related symptoms in a majority of all patients [40,44,48,56,57,58,78,79,80,81,82]. The bi-directional effect of these drugs on both MC survival/expansion and MC activation is remarkable and of great importance and may be useful in the management of symptomatic patients, following the principles of personalized medicine. It is worth noting in this regard that midostaurin has been reported to suppress IgE-dependent secretion of histamine in MC and basophils [83,84]. On the other hand, both drugs may also provoke certain side effects. For example, cladribine can induce cytopenia, immunosuppression and exanthemas [79,80,81], and midostaurin intake is often accompanied by nausea and vomiting [44]. 

For patients in whom no *KIT* D816V is detected, substantial cytoreduction (debulking) may also be achieved using imatinib or masitinib [42,43,45,47,85,86]. In rare cases, IFN-A may be considered and may induce clinically meaningful responses in slowly progressing ASM patients [74,87,88,89,90]. When the patient has at least a stable disease, is not eligible for HSCT or refuses HSCT, these drugs are maintained as long as the patient responds [14,44,47]. For patients who are resistant or have rapidly progressing advanced SM, poly-chemotherapy (poly-CT) is usually recommended using drugs and schedules otherwise administered in high risk acute myeloid leukemia (AML) [3,14,46,53]. These patients receive a nucleoside-type drug and high-dose ARA-C (FLAG or CLAG protocol) often in combination with midostaurin. Another option is to apply gemtuzumab-ozogamicine (GO) with poly-CT (instead of midostaurin), especially when the dominant sub-clone(s) or the entire disease lacks *KIT* D816V but expresses CD33 [46]. In each case and independent of the progression-status, HSCT should be considered as soon as the patient shows a good response to debulking therapy [59,77,78]. Sometimes, the initially poor performance status that precluded the patient from HSCT, improves dramatically with cladribine, midostaurin and/or poly-CT, so that the patient can then undergo HSCT. 

In older and/or unfit patients, long-term treatment with cladribine or midostaurin should be considered with recognition of side effects. Palliative treatment with hydroxyurea (HU) is offered in treatment-resistant cases [78]. In patients with SM-AHN, separate treatment plans for the SM component of the disease and AHN component of the disease should be established [2,3,6,9,14,78]. Sometimes, only the AHN portion requires intensive (hematologic) therapy in these patients (example: ISM-AML). 

The only curative approach for patients with advanced SM remains allogeneic HSCT [59,77]. The overall outcome of HSCT in advanced SM is better in patients who did not (yet) progress to MCL and those who have ISM-AHN or ASM compared to MCL [59]. Moreover, survival is better in patients who received myeloablative conditioning compared to dose-reduced conditioning prior to HSCT [59]. Whether post-HSCT therapy with KIT inhibitors, such as midostaurin, has a beneficial effect on long-term (progression-free) survival is currently unknown. However, it seems reasonable to apply midostaurin post-HSCT to suppress residual *KIT*-mutated clones in ASM/MCL patients [48].

In ISM-AHN, the situation is different. Here, the AHN (e.g., AML) must be eradicated but the HSCT is usually unable to also eradicate the ISM-component of the disease, presumably because MC and their progenitors are largely dormant (non-cycling) and thus resistant to conditioning regimens. However, this is acceptable and does not need post-HSCT therapy. In fact, these patients are often cured from their AHN (e.g., AML) and the residual ISM in their BM is not relevant clinically (P.V. and W.R.S., personal observation). 

## 6. Management of Mediator-Related Symptoms, Anaphylaxis and MCAS

In a considerable number of patients with CM and SM, recurrent systemic mediator-related symptoms develop [2,3,4,5,6,30]. However, in other patients with MC disorders, no symptoms are recorded, even when followed over a longer time period. Mediator-induced symptoms may be mild, moderate, substantial or even life-threatening. The treatment aims are to avoid any symptomatology by prophylactic use of anti-mediator-type medicines and to suppress mediator-related symptoms once these occur as much as possible [2,3,4,5,6,14,63,66,67,68,69,90,91]. 

Therefore, all patients with CM and SM are advised to stay away from (avoid) any potential triggers that may provoke MC activation and thus clinical symptoms [14,63,66,67,68,69]. High risk patients are advised to carry epinephrine self-injector-pens and to avoid any situation in which they may be alone and far away from any medical support-system (hospital, doctor). In addition, all symptomatic patients are advised to avoid histamine-rich foods and drinks. Finally, all patients with SM should receive prophylactic histamine receptor (HR) blockers, including HR1 and HR2-targeting drugs (Table 5) [14,63,66,67,68,69,90,91]. 

Additional treatment of symptoms in patients with CM or SM depends on the organ system involved, the severity of the reactions and the presence of co-morbidities. In patients with major gastrointestinal (GI) symptoms, higher doses of HR2 blockers may be required. In case of resistant (e.g., ulcerative) disease, the addition of a proton pump inhibitor is usually recommended (but the HR2 blocker must never be stopped) (Table 5) [14,63,67,90]. When these drugs cannot induce relief, the physician must consider alternative differential diagnoses and ask for a repeated endoscopic investigation of the GI tract. Some of these patients may benefit from treatment with low dose glucocorticosteroids and/or cromolyn sodium [92]. Skin symptoms, like pruritus or flushing, are initially treated with HR1 blockers. If this does not work, low dose systemic or topical glucocorticosteroids may be applied. Sometimes the skin lesions may also respond to ketotifen or to PGD2 receptor inhibitors. However, all these drugs are unable to eradicate the pigmented skin lesions seen in CM and SM. Whether the novel KIT-targeting tyrosine kinase inhibitors (TKI), such as midostaurin or avapritinib, can suppress or even eliminate skin involvement in CM and SM remains at present unknown. The first clinical observations suggest that skin lesions may indeed decrease and sometimes even fade away under such TKI therapy [56]. Ultraviolet A (UVA) therapy can also suppress skin lesions in SM [93,94]. However, after treatment discontinuation, the skin lesions always re-appear. 

In patients with IgE-dependent allergic reactions, therapy must effectively suppress mediator-induced symptoms. In these patients, treatment with systemic glucocorticosteroids may be required (Table 5). In addition, specific immunotherapy should be considered where possible [14,63,64,65,66,67,68,69,95]. In patients with massive, IgE-dependent hypersensitivity reactions to certain antigens, like bee or wasp allergens, specific immunotherapy is standard and should be performed life-long [63,64,65,66,67,68,69]. If this does not work, additional experimental drug therapies such as IgE-depletion (e.g., with omalizumab) must be considered [63,64,65,66,67,68,69,96]. These patients usually suffer from a mixed (primary and secondary) form of MCAS and are at the highest risk concerning life-threatening anaphylactic events.

An important aspect is the possible influence of neurological, psychological, mental or psychiatric problems on mediator-dependent symptomatologies in patients with mastocytosis [97,98]. These patients may need additional expert advice and support in order to control their symptoms. Whether these symptoms are primarily triggered by MC-derived mediators and/or are mainly caused by certain co-morbidities, remains at present unknown.

## 7. Management of Osteopathy in Mastocytosis—Current Standards

When the T-Score drops to below −2, therapy with bisphosphonates is initiated and the T-Score is re-examined every 6–12 months regardless of the type of SM, age, or co-morbidities [14,78]. In fact, these drugs are known to counteract bone loss in SM [99,100]. All patients with vitamin D deficiency should receive additional vitamin D (Table 5). When high vitamin D doses are required to correct the deficiency, additional vitamin K2 should be considered to avoid any risk of atherosclerosis. When bisphosphonate therapy fails, RANKL inhibitors and/or low-dose IFN-A can be considered (Table 5) [101,102,103,104]. Some of these patients may respond to combinations of IFN-A and bisphosphonates or IFN-A and a RANKL inhibitor. Whenever possible, systemic glucocorticosteroids (known to trigger osteoporosis) should be avoided in such patients.

## 8. Summary and Future Perspectives

Mastocytosis is a complex disease with diverse clinical manifestations and a variable clinical course. In non-advanced diseases, patients are mainly suffering from the cosmetic consequences of skin involvement, from symptoms produced by MC-derived mediators and/or from osteopathy. In advanced SM, additional problems have to be addressed, such as SM-induced organ damage and/or progression of the disease to secondary leukemia. The complex molecular mechanisms, multi-organ involvement, and the variable clinical course require a multi-disciplinary diagnostic approach and a multi-disciplinary treatment strategy that is ideally based on the principles of personalized medicine. The management of non-advanced patients is often based on symptom control and on the elimination or control of risk factors and co-morbidities. In addition, these patients may need psychological or psychiatric support. In patients with mediator-related symptoms, strict avoidance of any MC-activating triggers and avoidance of histamine-rich food/drinks is also of great importance. Independent of the SM variant, all patients receive prophylactic histamine receptor (HR1 + HR2) blockers. In those with substantial osteopenia or osteoporosis, bisphosphonates are usually prescribed. In advanced SM, the major question in the beginning is whether the patient is a candidate for allogeneic HSCT. If this is not the case, the major aim is to achieve long-term disease control by administration of cytostatic drugs, KIT-targeting drugs or immuno-modulating agents. Otherwise, these patients are prepared for HSCT. In each case, the treatment plan should be established in a multidisciplinary fashion and in collaboration with (or in) a center of excellence of mastocytosis. Based on knowledge accumulated in recent years about molecular markers and targets, the impact of co-morbidities and the mechanisms that underlie the disease evolution, personalized medicine approaches can now indeed be applied in mastocytosis patients in daily practice. In the future, many more molecular targets will be identified and will increase the panel of drug candidates and the therapeutic armamentarium in patients with SM. Overall, the authors believe that precision medicine and personalized medicine approaches will improve management and the quality of life in patients with mast cell neoplasms in the near future.

## Figures and Tables

**Table 1:** Clinical and laboratory features that argue for a bone marrow examination in adult patients with suspected mast cell disease.

--------------------------------------------------------------------------------------------------------------- Absolute indications: Typical skin lesions (+/− biopsy) suggesting mastocytosis in the skin Elevated basal serum tryptase level (>20 ng/mL or >30 ng/mL as isolated finding *) that is not based on a known familial hypertryptasemia or other myeloid neoplasm Expression of *KIT* D816V in circulating blood leukocytes or in skin-derived cells Mast cell activation syndrome patients in whom the event-free serum tryptase level is clearly elevated (>20 ng/mL)—with or without known familial hypertryptasemia Unexplained splenomegaly and/or lymphadenopathy Unexplained blood count abnormalities, e.g., eosinophilia and/or cytopenia Relative indications: ** Repeated anaphylactic shock after bee or wasp stings (+/− known allergy) Unexplained (idiopathic) mast cell activation syndrome Unexplained osteoporosis—especially in men Non-specific colitis and/or unexplained hepatopathy with ascites Histamine—symptomatology, e.g., otherwise unexplained headache + diarrhea Unexplained weight loss Unexplained gastrointestinal symptoms responding to HR2 blockers Unexplained elevated serum alkaline phosphatase activity Psychological/psychiatric instability ***

* When familial hypertryptasemia has been ruled out, a basal serum tryptase level exceeding 30 ng/mL is an indication for a bone marrow examination even if no further relevant laboratory parameters, signs or symptoms can be documented. When such additional features (like for example blood count abnormalities) are also found or the basal tryptase level increases, a value above 20 ng/mL is also sufficient to ask for a bone marrow examination. ** Relative indications alone may not necessarily lead to a bone marrow examination but in the presence of multiple indicators, a bone marrow investigation is required. *** In many patients with non-specific symptoms, it turns out that the underlying condition is a psychological issue or a psychiatric disease. When this possibility has been ruled out, the likelihood for the presence of a mast cell disease and/or a mast cell activation disorder increases. Abbreviations: HR2, histamine receptor 2.

**Table 2:** Classification of mastocytosis *.

------------------------------------------------------------------------------- **1. Cutaneous mastocytosis (CM)** Maculopapular cutaneous mastocytosis (MPCM) (= Urticaria pigmentosa = UP) Diffuse cutaneous mastocytosis (DCM) Mastocytoma of skin **2. Non-advanced systemic mastocytosis** Indolent systemic mastocytosis (ISM) Bone marrow mastocytosis (BMM) Smoldering systemic mastocytosis (SSM) **3. Advanced systemic mastocytosis** Aggressive systemic mastocytosis (ASM) ASM without signs of transformation ASM in transformation to MCL (ASM-T) Systemic mastocytosis with an associated hematologic (non-mast cell) neoplasm (SM-AHN) SM-AHN with myeloid neoplasm SM-AHN with lymphoid neoplasm Mast cell leukemia (MCL) Primary (de novo) MCL vs. secondary MCL Typical MCL vs. aleukemic MCL ** Acute MCL vs. chronic MCL *** 4. Mast cell sarcoma (MCS) -------------------------------------------------------------------------------

* The classification of mastocytosis relates to the proposal of the World Health Organization (WHO). ** In aleukemic MCL, circulating mast cells comprise <10% of all circulating leukocytes. *** In chronic MCL, no overt organ damage (C-Finding) is found.

**Table 3:** Relevant co-morbidities in patients with systemic mastocytosis (SM).

--------------------------------------------------------------------------------------------- **Relevant concerning survival:** Cardiovascular co-morbidities Metabolic diseases and syndromes Non-hematologic cancer **Relevant concerning allergy and anaphylaxis:** IgE-mediated allergy Chronic bronchitis Chronic inflammatory bowel disease Chronic autoimmune processes Chronic nicotine abuse Chronic alcohol abuse **Relevant concerning quality of life and mental status:** Psychiatric diseases Psychological problems Neurological disorders Mental problems Family problems and other private issues Food intolerance Drug reactions, drug intolerance **Relevant concerning osteopathy/osteoporosis leading to bone fractures:** Vitamin D deficiency Co-morbidities requiring chronic treatment with corticosteroids Co-morbidities requiring immobilization Gynecological disorders affecting bone density Endocrinological disorders affecting bone density Nephrologic diseases affecting bone density Obesity * --------------------------------------------------------------------------------------------

* Obesity itself is a risk factor for osteoporosis when leading to immobility. However, obesity is also a risk factor for (e.g., vertebral) bone fractures.

**Table 4:** Risk factors in patients with systemic mastocytosis (SM).

---------------------------------------------------------------------------------------- **Risk factors concerning shorter survival** Age Co-morbidities (cardiovascular, cancer, others) Advanced SM (ASM, ASM-T, MCL) Percent of mast cells in bone marrow smears (≥5%; ≥20%) Percent of myeloblasts and metachromatic blasts Associated hematologic neoplasm (AHN) Somatic mutations, especially in: *SRSF2*, *ASXL1*, and *RUNX1* *KIT* D816V allele burden Alkaline phosphatase level Splenomegaly and/or lymphadenopathy **Risk factors for disease progression to a higher grade SM variant** Advanced SM (ASM, ASM-T, MCL) Percent of mast cells in bone marrow smears (≥5%) Percent of myeloblasts and metachromatic blasts Associated hematologic neoplasm (AHN) Chromosomal abnormalities Somatic mutations, especially in: *SRSF2*, *ASXL1*, and *RUNX1* *KIT* D816V allele burden Multilineage involvement with *KIT* D816V Increasing alkaline phosphatase level Splenomegaly and/or lymphadenopathy Elevated β2-microglobulin Absence of skin lesions Steadily increasing basal serum tryptase level Elevated (increasing) interleukin-6 levels **Risk factors for anaphylaxis and MCAS** Known IgE-dependent allergy Known atopic disorder (with high IgE level) Anaphylactic reactions in the case history Adverse reactions after bee or wasp stings Patient not agreeable to take prophylactic drugs Drug or food intolerance Histamine-related symptomatology Prior exposure to allergens (skin test) ----------------------------------------------------------------------------------------

ASM, aggressive systemic mastocytosis; ASM-T, ASM in transformation to MCL; MCL, mast cell leukemia; MCAS, mast cell activation syndrome; IgE, immunoglobulin E.

**Table 5:** Precision medicine-based therapy in patients with systemic mastocytosis (SM).

---------------------------------------------------------------------------------------------------------------	
**Condition/indication**	**Recommended therapy**
---------------------------------------------------------------------------------------------------------------	
Anaphylaxis/hypotension	1. HR1+HR2 blocker (basic therapy) 2. Glucocorticosteroids 3. Specific immunotherapy (known bee or wasp allergy) 4. Omalizumab (IgE-dependent allergy)
Confirmed involvement of	Aspirin * + HR2 blocker
arachidonic acid derivatives (PGD2)	
Severe anaphylaxis/MCAS	Omalizumab

GI-tract problems	
Ulcerative GI tract disease	1. Appropriate doses of HR2 blocker
Resistant ulcerative GI tract disease	2. Proton pump inhibitors + HR2 blocker
Crampi, constipation, loose stools	HR2 blocker
Chronic diarrhea	Appropriate doses of HR2 blocker
With dense mast cell infiltrates	Consider cytoreductive therapy (when C-Findings are recorded)
With ascites and hepatopathy	Consider cytoreductive therapy (C-Finding fulfilled)

Osteopenia/Osteoporosis	
Progressing osteopenia	Bisphosphonates when T-score < −2
Osteopathy with vitamin D deficiency	plus Vitamin D (+/− vitamin K2 **)
Osteoporosis (T Score < −2)	Bisphosphonates
Resistant osteoporosis	plus RANKL inhibitor and/or plus low dose interferon-alpha

Skin involvement in SM	HR1 blocker
Severe/resistant skin symptoms	plus glucocorticosteroids (systemic/topical) and/or UVA or PUVA therapy

Disease progression without AHN	
* KIT* D816V+ ASM with slow progression *	Cladribine, midostaurin, IFN-A
* KIT* D816V− ASM with slow progression *	Imatinib, masitinib, midostaurin
ASM with rapid progression or MCL	Polychemotherapy + HSCT
ASM or MCL not eligible for HSCT	
or not willing to have a HSCT	Cladribine, midostaurine, IFN-A
Palliative management	HU, midostaurin, BSC
Disease progression with/to AHN	
ASM-AHN or MCL-AHN	Separate treatment plans:
or ISM-AHN	treat the AHN portion of the disease as if no SM was diagnosed and SM portion as if no AHN was found
---------------------------------------------------------------------------------------------------------------	

* Aspirin is not recommended for patients with GI tract disease or a high risk of development of an ulcerative GI disease. In addition, aspirin may provoke idiosyncratic reactions and severe hypotension. Note also that relatively high doses of aspirin (500 mg/day or more) are required to suppress prostaglandin synthesis in mast cells in patients with mastocytosis. ** In young and fit patients who are eligible, HSCT must be considered, independent of the response to initial therapy. In those who respond well to interventional therapy, no HSCT may be required or may be delayed. Abbreviations: HR, histamine receptor; IgE, immunoglobulin E; MCAS, mast cell activation syndrome; PGD2, prostaglandin D2; MCAS, mast cell activation syndrome; GI tract, gastrointestinal tract; UVA, ultraviolet light; AHN, associated hematologic (non-mast cell) neoplasm; ASM, aggressive systemic mastocytosis; MCL, mast cell leukemia; IFN-A, interferon-alpha; HSCT, hematopoietic stem cell transplantation; HU, hydroxyurea; BSC, best supportive care.

## Supplementary Materials

Supplementary materials can be found at https://www.mdpi.com/1422-0067/20/12/2976/s1.

## Abbreviations

HR	histamine receptor
IgE	immunoglobulin E
MCAS	mast cell activation syndrome
PGD2	prostaglandin D2
GI tract	gastrointestinal tract; UVA, ultraviolet light
AHN	associated hematologic (non-mast cell) neoplasm
ASM	aggressive systemic mastocytosis
MCL	mast cell leukemia
IFN-A	interferon-alpha
HSCT	hematopoietic stem cell transplantation
HU	Hydroxyurea
BSC	best supportive care
