# Supplementary material for: Multidisciplinary Challenges in Mastocytosis and How to Address with Personalized Medicine Approaches
Source: Int J Mol Sci. 2019 Jun 18;20(12):2976. doi: 10.3390/ijms20122976 (PMC6627900; doi:10.3390/ijms20122976)
Supplement: Supplementary file 1 [file ijms-20-02976-s001.pdf]

Supplemental Material to Valent et al:

Multidisciplinary Challenges in Mastocytosis and how to address with Personalized Medicine Approaches

Supplemental Tables

Supplemental Table S1

Major and Minor WHO Criteria of Systemic Mastocytosis (SM criteria)*

Major criterion:	Multifocal dense infiltrates of mast cells (>15 mast cells in aggregates) in bone marrow biopsies and/or in sections of other extracutaneous organ(s)
Minor criteria:	a. >25% of all mast cells are atypical cells (type I or type II) on bone marrow smears or are spindle-shaped in mast cell infiltrates detected on sections of visceral organs
	b. *KIT* point mutation at codon 816 in the bone marrow or another extracutaneous organ
	c. mast cells in bone marrow or blood or another extracutaneous organ expresses CD2 or/and CD25
	d. Baseline serum tryptase concentration >20 ng/ml (in case of an unrelated myeloid neoplasm, d. is not valid as an SM criterion)

If at least one major and one minor or three minor criteria are fulfilled
→ the diagnosis is systemic mastocytosis = SM

*SM criteria have been defined by the WHO in 2001 and confirmed in 2008 and 2016.
Abbreviations: WHO, World Health Organization; SM, systemic mastocytosis.

Supplemental Table S2

WHO Classification of Mastocytosis 2016*

Cutaneous mastocytosis (CM)
- Maculopapular CM (MPCM) = urticaria pigmentosa (UP)
- Diffuse CM (DCM)
- Mastocytoma of skin

Systemic mastocytosis (SM)
- Indolent SM (ISM)
- Smoldering SM (SSM)**
- SM with associated hematologic (non-mast cell) neoplasm (SM-AHN)
- Aggressive SM (ASM)
- Mast cell leukemia (MCL)

Mast cell sarcoma

---

*The WHO classification of mastocytosis was established in 2001 and was confirmed in 2008 and 2016, with minor modifications.
**The smoldering type of SM (SSM) was initially regarded as a provisional sub-category of ISM. Later, in 2007, SSM was recognized as separate category of SM by the EU-US consensus group.
Abbreviations: WHO, World Health Organization;
EU-US, European - United States of America.
